# Multicentric Castleman's Disease, Associated with Idiopathic Thrombocytopenic Purpura

**DOI:** 10.1155/2013/269268

**Published:** 2013-10-02

**Authors:** Ruchi Sood, Harris C. Taylor, Hamed Daw

**Affiliations:** ^1^Fairview Hospital, Cleveland Clinic Health System, Cleveland, OH 44111, USA; ^2^Case Western University School of Medicine, Cleveland, OH 44111, USA; ^3^Hematology-Oncology, Fairview Hospital, Cleveland Clinic Health System, Cleveland, OH 44111, USA

## Abstract

The most common cause of a neck mass in young adults is hyperplastic lymphadenopathy consequent to infection and inflammation. Castleman's disease (CD), an unusual benign lymphoproliferative disorder, infrequently causes neck masses. It occurs in unicentric (UCD) and multicentric (MCD) forms and is associated with human immunodeficiency virus (HIV), human herpes virus 8 (HHV-8), and Kaposi's sarcoma. We present the third known association between MCD and previous immune thrombocytopenia in the absence of HIV and HHV-8 infection and review its association with other autoimmune disorders and attendant implications for pathogenesis. Finally, we summarize the current approach to therapy.

## 1. Case Presentation

A 27-year-old female with a history of asthma, presented with diffuse ecchymoses and nosebleeds in 2006. Her platelet count was as low as 3 × 10^9^/L having decreased from 112 × 10^9^/L in 2005. Her white cell count was 7.5 × 10^9^/L and hemoglobin was 15.2 g/dL. HIV testing was negative, and no new medications had been started. Because of the severe isolated decline in platelet count, her thrombocytopenia was thought to be immune thrombocytopenia. She was begun on intravenous steroids and immunoglobulins. All signs of bleeding resolved but the platelet count started to drop while she was on the prednisone taper. She eventually required splenectomy after which her platelets increased to 461 × 10^9^/L. Her histological findings were compatible with immune thrombocytopenia. On follow-up visit 5 months later, she presented with significant episodes of dental bleeding and bruising and her platelet count had decreased to as low as 2 × 10^9^/L. She was started on rituximab only chemotherapy after which she remained in complete remission.

**Figure 1:**
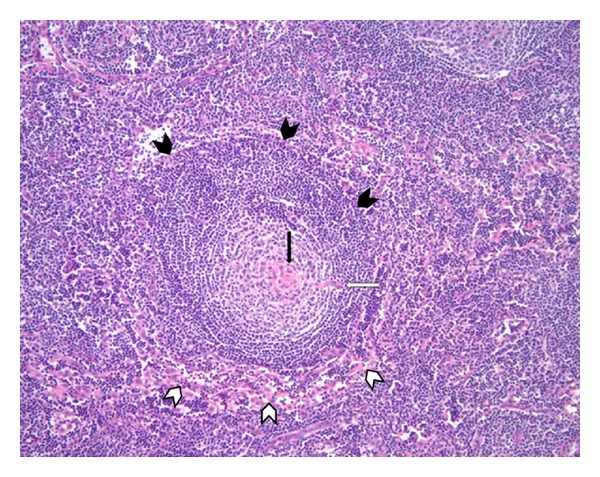
Marked angiofollicular hyperplasia representing hyaline vascular variant. Concentric “onion skin pattern” of peripheral lymphocytes (black arrow heads) surrounding a pale centered follicle (thin black arrow). Vascular proliferation (white arrow heads) is seen with capillaries demonstrating thickened walls and prominent endothelial cells. Penetrating hyalinized small vessel gives follicle a lollipop-like appearance (thin white arrow).

**Figure 2:**
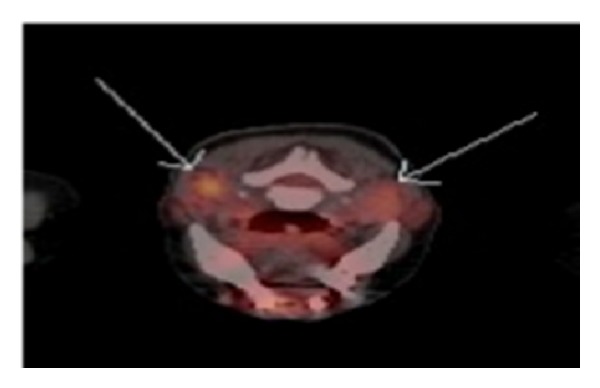
Before treatment: PET scan at the base of tongue, showing lingual adenopathy (White arrows).

**Figure 3:**
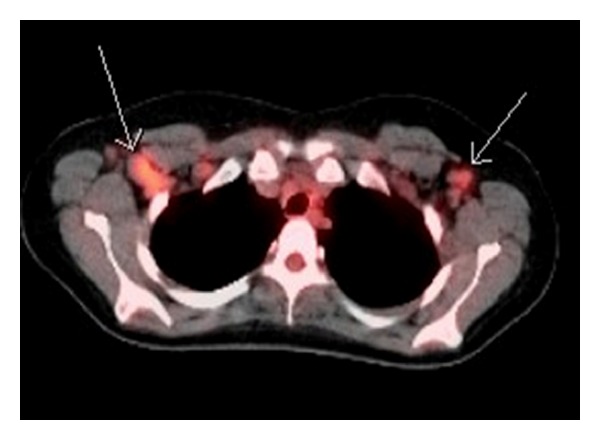
Before treatment: whole body scan with cut through the level of the lungs, showing axillary lympadenopathy (White arrows).

In March 2012, she presented with a three-month history of a growing, right lower neck mass. She denied any history of bleeding, fever, chills, night sweats, or weight loss. Examination revealed an afebrile female with hard, fixed, painless, right cervical lymphadenopathy measuring 4 × 2 cm. There were no other palpable lymph nodes. WBC was 11.23 × 10^9^/L (34% segmented neutrophils, 49% lymphocytes, 5% reactive lymphocytes, 7% monocytes, 5% eosinophils, and 0% basophils), Hgb 12.7 g/dL, and platelets 415 × 10^9^/L. Her comprehensive metabolic panel, serum protein electrophoresis, and lactate dehydrogenase were unremarkable. Pathology of the excised node revealed marked angiofollicular hyperplasia consistent with the hyaline vascular variant of CD (Figure 1). Contrast enhanced CT scan of the chest and abdomen showed bilateral axillary adenopathy of approximately 3 cm and mild bilateral superficial inguinal adenopathy. PET scan showed intense activity at the base of the tongue, consistent with lingual adenopathy, causing significant mass effect on the oropharyngeal airway. SUV max was 8.2 (Figure 2). There were numerous enlarged bilateral jugular, axillary, subcarinal, hilar, and inguinal lymph nodes. The right pelvic wall and parts of the stomach, large and small intestine also demonstrated mild to moderate fluorodeoxyglucose (FDG) uptake consistent with MCD (Figure 3). HIV antibodies and Western Blot were negative as was HHV-8 testing by immunohistochemistry and PCR. Serum IgG was 1065 mg/dL, IgA 366 mg/dL, and IgM 122 mg/dL. 

**Figure 4:**
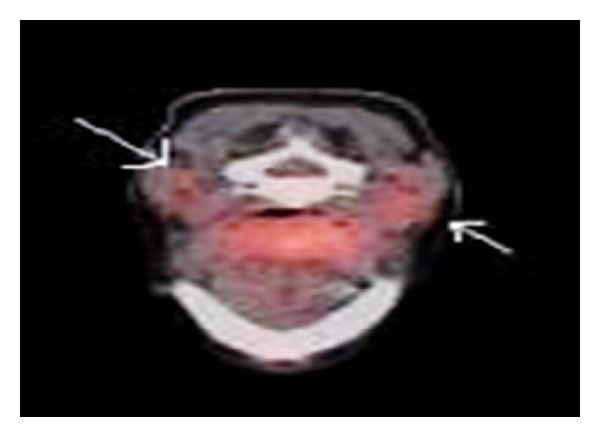
After treatment: PET scan at the base of tongue, showing resolution of previous noted lingual adenopathy (White arrows).

**Figure 5:**
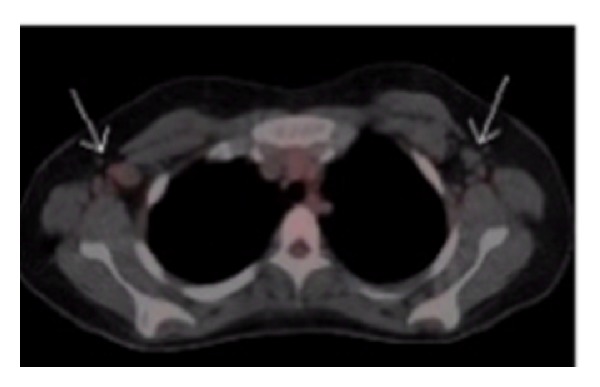
After treatment: whole body scan with cut through the level of the lungs, showing resolution of previously noted axiallary lymphadenopathy (White arrows).

She was started on rituximab chemotherapy (375 mg/m^2^) intravenously once weekly for 4 doses. Repeat PET scan done three months later showed decreased uptake at the base of tongue with an SUV max of 6.1 (Figure 4). Likewise, significant decrease in uptake of FDG was seen in the bilateral jugular, axillary, subcarinal, and hilar nodes as well as the right pelvic wall, small and large bowel (Figure 5). 

Her last followup was seven months later, and she was found to be asymptomatic. 

## 2. Discussion

CD is also known as angiofollicular lymph node hyperplasia, giant lymph node hyperplasia, angiomatous lymphoid hamartoma, lymph node hamartoma, and benign giant lymphoma. The first case report of MCD was published in 1954 [1]. Two years later, Castleman described 13 patients with unicentric hyaline vascular CD of the chest [2]. Since it is a rare disease, there is no reliable information on its incidence [3]. It typically presents as a mediastinal mass and primarily involves the lymphatic tissue [4]. Extralymphatic sites of involvement include the lungs, larynx, parotid glands, pancreas, meninges, and muscles [5].

There are two major histopathogenetic types, hyaline vascular (HV) and the plasma cell (PC) variant. An intermediate form has also been described [6]. The HV type, which represents 90% of the cases, is characterized by follicular abnormalities and abundant interfollicular vascularization. The PC variant is characterized by hyperplastic germinal centers with plasma cell infiltration in the intervening areas. 

The HV is considered to be an early form of the disease, whereas the PC variant is considered to be a mature form of the disease and is believed to be related to a stronger immunological response [7].

UCD, the less aggressive form of the disorder, presents as a solitary lesion. It is usually seen in young and middle aged patients without any sex predilection. The HV type is unicentric in 90% of the cases [8]. In contrast, MCD presents as generalized lymphadenopathy and is frequently associated with hepatosplenomegaly and constitutional symptoms. Anemia, hypoalbuminemia, and hypergammaglobulinemia are also common [9]. It is strongly associated with immunosuppression as in HIV and HHV-8 infection [10]. MCD ordinarily occurs in the fourth or fifth decades but may occur at younger ages in people who are HIV positive [11]. The PC variant is the predominant pathologic pattern in this type.

The etiology of CD remains unknown although both immunodeficiency and autoimmunity have been proposed. Chronic inflammation resulting from exposure to an unknown antigen has been supported by the presence of excessive serum levels of interleukin -6 (IL-6), a cytokine with pleiotropic effects on the immune system and hematopoiesis. Some believe it may play a central role in the pathophysiology of CD [12–14]. The association of CD with autoimmune disorders including myasthenia gravis [15], Evans' syndrome [16], vitiligo [17], coeliac disease [18], Graves' disease [19], and ulcerative colitis [20] suggests a possible autoimmune pathology. Of considerable interest, there are two previous reports of the association between CD and immune thrombocytopenia [21, 22]. However, it is not clear if autoimmunity is the underlying cause or result of CD [23]. Since our patient had a previous underlying autoimmune disorder in the form of immune thrombocytopenia, it appears as if an autoimmune response may be the cause in our patient. However, the possibility that this represents a chance association should be acknowledged.

The mainstay of treatment for UCD is complete surgical resection. Rarely, local recurrence has been reported [24]. In the case of partially resected lesions, the outcome is favorable, since patients may remain asymptomatic for years. Radiotherapy is the best option when surgical resection is not possible [25]. For MCD, complete surgical debulking is rarely possible. There is no definitive treatment as no randomized controlled trials have been performed due to its rarity. The available literature on MCD therapy consists mainly of single case reports and case series. Rituximab, a monoclonal anti-CD 20 antibody, has significant activity against both HIV positive and negative MCD, even when used as salvage therapy. It was first introduced for the treatment of HIV positive patients with MCD after chemotherapy-induced remission [26].

Most MCD cases treated to date with rituximab have been HIV and HHV-8 positive patients. To our knowledge, approximately seven HIV negative patients with MCD have been treated with rituximab [26–33]. Rituximab was chosen in our patient as the first-line treatment since she also had a history of immune thrombocytopenia.

## 3. Conclusion

MCD is usually seen in the fourth and fifth decades of life. It is uncommon to encounter in a 27-year-old female in the absence of HIV or HHV-8 infection. Although infrequent, it is well known that hematological abnormalities, including anemia, leukopenia, or thrombocytopenia, can be encountered during the clinical course of CD [34–36]. However, the presence of CD during the clinical course of immune thrombocytopenia has been rarely described [21, 22].
